# Prognostic role of delta radiomics in pediatric pontine diffuse midline gliomas

**DOI:** 10.1007/s11060-026-05457-y

**Published:** 2026-02-11

**Authors:** Erhan Bıyıklı, Tahsin Aybal, Bulent Aslan, Dilek Gul, Nursah Eker, Beste Melek Atasoy, Ayse Gulnur Tokuc, Feyyaz Baltacıoglu

**Affiliations:** 1 Department of Radiology, Marmara University School of Medicine, 10 Muhsin Yazicioglu St., Istanbul, 34899 Turkey; 2 Department of Radiology, Kartal Lütfi Kırdar City Hospital, D-100 South Side Road, No:47, Istanbul, 34865 Turkey; 3 Department of Radiology, Beth Israel Deaconess Medical Center, 330 Brookline Ave, Boston, MA 02215 USA; 4 Department of Radiation Oncology, Marmara University School of Medicine, 10 Muhsin Yazicioglu St., Istanbul, 34899 Turkey; 5 Department of Pediatric Oncology, Marmara University School of Medicine, 10 Muhsin Yazicioglu St., Istanbul, 34899 Turkey; 6 Department of Radiology, American Hospital, Guzelbahce St., Istanbul, 34365 Turkey

**Keywords:** Diffuse midline glioma, Delta radiomics, Prognosis, Texture analysis, Imaging biomarkers

## Abstract

**Purpose:**

Pontine diffuse midline gliomas (PDMGs) are among the most lethal pediatric brain tumors with a median survival of approximately one year. Reliable prognostic markers are needed to guide treatment strategies and inform families. Our study aims to investigate the prognostic utility of delta-radiomic features in PDMGs.

**Materials and methods:**

We retrospectively analyzed 35 pediatric patients with PDMG diagnosed between 2012 and 2020, all treated with radiotherapy plus concomitant and adjuvant temozolomide. Pre- and post-treatment MRI (T1-weighted, T2-weighted, and ADC maps) were subjected to manual segmentation and texture feature extraction using MaZda software. Delta radiomics features were calculated as ratios between post- and pre-treatment values. Diagnostic performance for predicting survival below or above 12 months was assessed using ROC analysis, while overall survival was further evaluated with Kaplan–Meier and Cox regression models.

**Results:**

Median overall survival was 13 months (range: 6–69). Among baseline features, only sum average from non-contrast T1 was significant, with lower values (indicating higher heterogeneity) associated with shorter OS. In the post-radiotherapy setting, sum entropy from T1 images emerged as an independent prognostic predictor, with higher values correlating with longer OS. Delta radiomics parameters, particularly sum entropy derived from T1 and T2 sequences, yielded higher AUC values than single post-treatment features, suggesting superior prognostic accuracy.

**Conclusion:**

Our preliminary results indicate that delta-radiomics outperforms static texture analysis in predicting overall survival in pediatric PDMGs, and to the best of our knowledge, this is the first study to investigate the prognostic utility of delta-radiomics in this population. Evidence is provided that delta radiomics can serve as a non-invasive marker for early prognostic stratification in pediatric PDMGs. Validation in larger, multi-center cohorts is required to confirm its clinical utility.

**Supplementary Information:**

The online version contains supplementary material available at 10.1007/s11060-026-05457-y.

## Introduction

Diffuse midline gliomas are most commonly located in the pons in children, accounting for approximately 15% of all pediatric brain tumors, which are classified as a CNS WHO grade 4 tumors irrespective of histological features [1]. In the pediatric population, pontine diffuse midline gliomas (PDMGs) are incurable and lethal tumors that do not respond to available treatments. These tumors carry a grim prognosis, with a median survival of 9 to 12 months after diagnosis [2]. Owing to the paucity of data on the superiority of targeted therapies, clinicians are frequently challenged in clinical practice to convey the rationale and potential contribution of biopsy to patients and their families [3, 4].

Conventional MRI findings related to poor prognosis contain cyst or necrosis, ring enhancement, diffusion restriction, disseminated disease and invasion of adjacent structures [5, 6],whereas these findings have not been employed to predict overall survival [7]. The use of radiomics has gained popularity over the past decades due to its non-invasive nature and its ability to quantify imaging features, thereby providing complementary information to visual assesment [8]. Radiomic features can be applied for non-invasive detection of mutation status such as H3K27M, ACVR1 and TP53 [8–10]. Moreover, several studies have demonsstrated MRI-based radiomics offers the opportunity to estimate overall survival [11–14]. 

Recently, the concept of “delta radiomics,” also termed “longitudinal texture analysis,” has been introduced to evaluate dynamic alterations in radiomic features obtained at multiple imaging time points, generally pre- and post-treatment, allowing assessment of therapy response and biologically driven changes [15, 16]. In the current literature, numerous studies have been conducted to assess treatment response, progression-free survival, or overall survival, with the majority focusing on lung, rectal, and gastrointestinal cancers [16]. Nevertheless, delta radiomics investigations in central nervous system tumors remain scarce, and the available studies have been predominantly performed in adult populations [17–19]. To date, no studies have explored whether delta radiomics features could be applied for prognostic prediction in pediatric PDMGs. Our hypothesis is that delta-radiomic features offer improved overall survival prediction over baseline-only models.

## Methods

### Study design and patient selection

This retrospective study was approved by the Ethical Committee of the School of Medicine for Non-Interventional Studies, and the requirement for informed consent was waived. All consecutive patients with a clinico-radiological diagnosis of PDMG at our institution between 2012 and 2020 were included, in accordance with the Declaration of Helsinki.

**Fig. 1:**
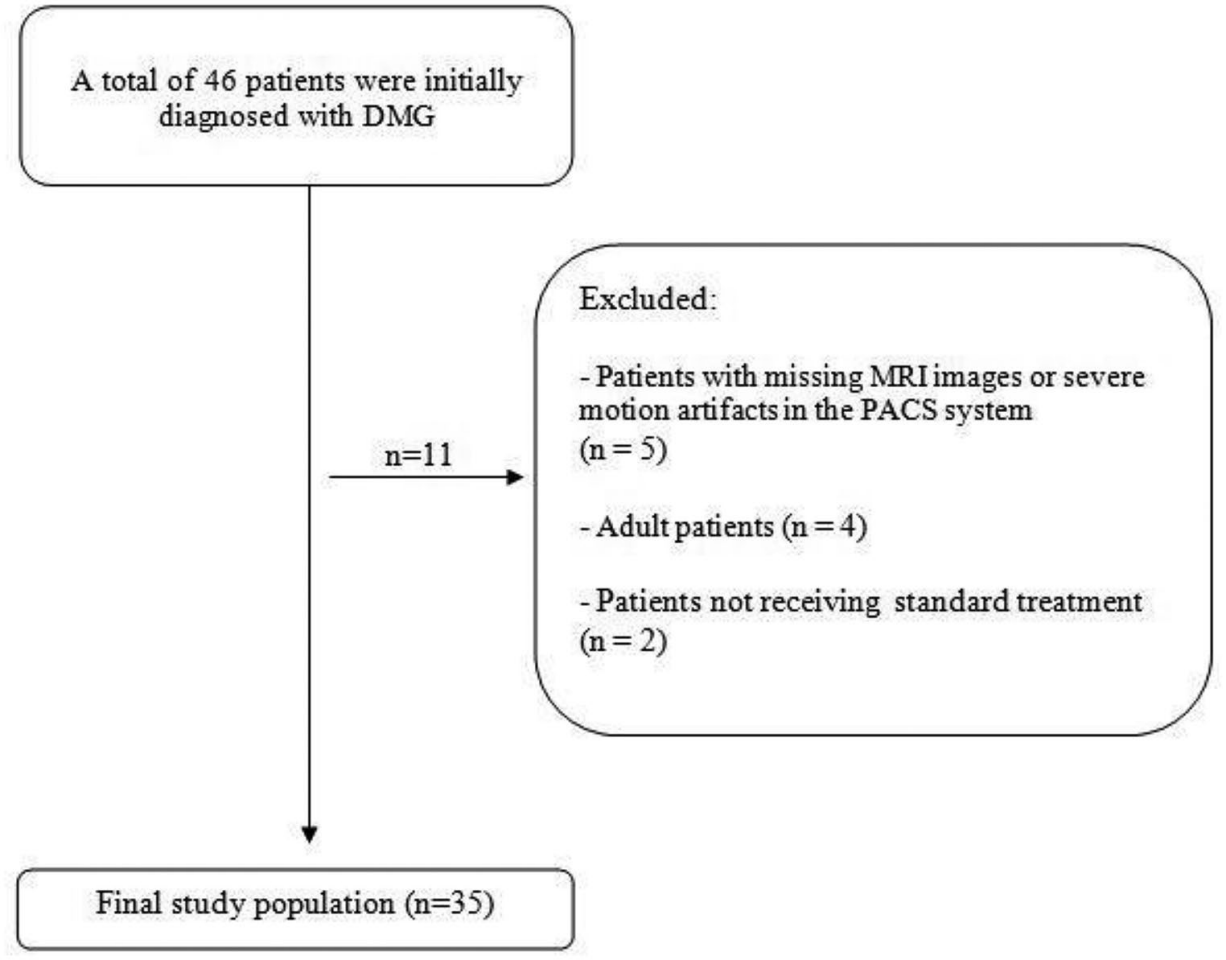
Flow chart of the study

A total of 46 patients were initially diagnosed with PDMG. Four adult patients were excluded based on age criteria, five patients were excluded due to the absence of MRI images in the PACS system or severe motion artifacts, and two patients were excluded because they did not receive standard therapy. Consequently, 35 pediatric patients with PDMG were included in the final analysis (Fig. 1). In line with previous literature, patients were stratified into two groups according to overall survival of less than 12 months (short survivors) and 12 months or more (long survivors). Overall survival (OS) was determined as the time from the diagnostic MRI scan to death from any cause.

All patients received radiotherapy using a linear accelerator with 6 MV photon energy, delivering 50.4 to 54 Gy in 28–30 fractions, along with concomitant (90 mg/m²/day for 42 days) and adjuvant temozolomide until clinical or radiological progression.

### MRI acquisition

All MRI scans were obtained with 1.5-T or 3-T scanners (Avanto and Verio, Siemens, Erlangen, Germany). Standard brain imaging protocol includes Axial T2-WI, Coronal FLAIR, DWI/ADC at b-values 0 and 1000, pre-contrast Axial T1-WI and 3D or axial post-contrast T1-WI. All MRI examinations included standard pre-contrast sequences consisting of T1-weighted spin-echo imaging (TR 450 ms, TE 10 ms, flip angle 90°, FOV 200–260 mm), T2-weighted spin-echo imaging (TR 4200 ms, TE 90 ms, flip angle 150°, FOV 200–260 mm), T2-FLAIR imaging (TR 7000 ms, TE 84 ms, flip angle 150°, FOV 200–260 mm), and axial diffusion-weighted imaging with b-values of 0 and 1000 s/mm², from which ADC maps were generated. Post-contrast T1-weighted imaging was acquired using either a 3D-TFE sequence (TR 7 ms, TE 3 ms) or an axial TSE sequence (TR 500 ms, TE 10 ms), depending on patient motion susceptibility. Pre- and post-treatment MRI scans were not consistently acquired on the same scanner; rather, scanner assignment occurred randomly based on clinical workflow.

### Texture analysis

**Fig. 2:**
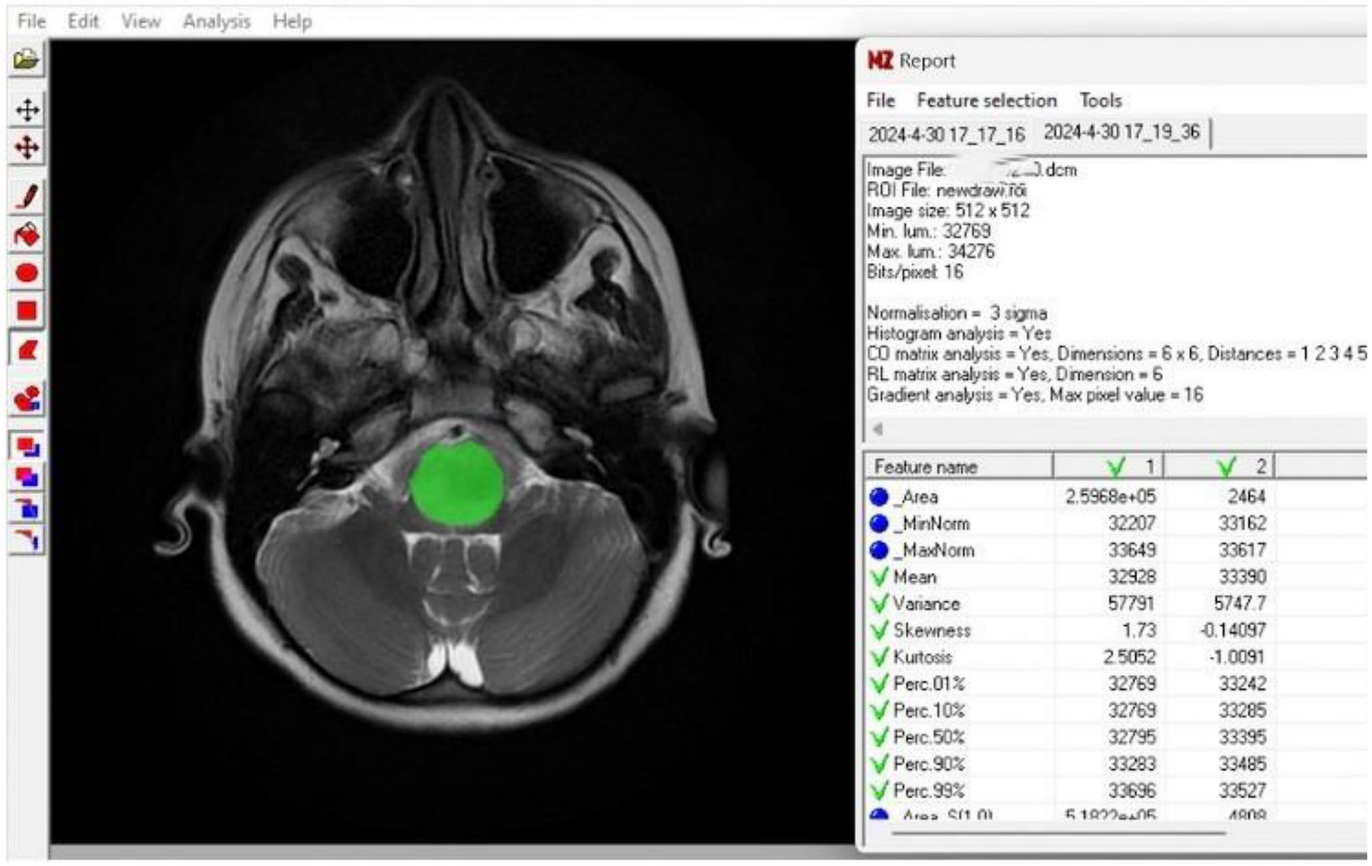
A patient’s measurement sample with the texture analysis program

MR texture analysis (MRTA) was performed using MaZda software (version 4.6, Institute of Electronics, Technical University of Łódź, Poland; http://www.eletel.p.lodz.pl/programy/mazda/). Images were exported in DICOM format for calculations. Pre and post-treatment (3 months after treatment) axial T2-WI, axial T1-WI and ADC map images were used to extract radiomic features. Post-contrast series were excluded from the analysis because of heterogeneity in imaging sequences; specifically, either 3D-TFE or axial TSE post-contrast acquisitions were used depending on the patient’s motion susceptibility, leading to variability that could affect radiomic feature stability. For each patient and sequence, regions of interest (ROIs) were manually delineated to encompass the entire solid part of tumor by a radiologist (B.A.) who was blinded to the clinicial information (Fig. 2). For standardization, image intensities were normalized using the µ ± 3σ method available in the software, and rescaled into 64 discrete gray levels. From the segmented ROIs, a set of first- and second-order texture features was extracted, including basic descriptors (area, mean, variance, skewness, kurtosis), as well as co-occurrence matrix–based parameters such as angular second moment, contrast, correlation, sum of squares, inverse difference moment, sum average, sum variance, sum entropy, entropy, difference variance, and difference entropy. In addition, delta features were derived as the ratio (post/pre) between pre- and post-treatment values. We selected texture features based on non-parametric statistical testing. Only the features that showed a statistically significant difference between the groups were included in the analysis.

### Statistical analysis

Calculations were performed using the SPSS v22.0 software (IBM Corp., Armonk, N.Y., USA). The Kolmogorov-Smirnov test was used to determine whether data was distributed normally. Median and minimum-maximum values were used for descriptive analysis. Non-parametric variables which did not have normal distribution were compared using the Mann Whitney U test. Kaplan-Meier and Cox regression analyzes were used for survival analyses. Diagnostic performance in predicting cases with survival under 12 months and 12 months or more was evaluated by ROC analysis. A confidence interval of 95% and p values below 0.05 were considered statistically significant.

## Results

Thirty five patients (21 males, 14 females) were included in this research. The median age was 8 years (range: 1–17). Four patients were alive at the time of analysis. The median overall survival was 13 months (range: 6–69).

When patients with a life expectancy of less than 12 months (short life expectancy) and those with a life expectancy of 12 months or more (long life expectancy) were evaluated as two separate groups, the statistically significant texture analysis parameters are shown in Table 1. Based on this distinction, patient age and other textural features outside Table 1 were not found to be statistically significant, and all extracted features are presented in Supplementary Table 1.

**Table 1:** Diagnostic success of statistically significant texture analysis and delta texture analysis parameters in distinguishing patients with a survival time of less than 12 months from those with a survival time of 12 months or more

Texture features	*p* value	AUC (CI)
SumAverg T1 Pre-treatment SumEntropy T1 Post-treatment Delta SumEntropy T1 Delta SumEntropy T2	**0.030** **0.007** **0.001** **0.005**	0.719 (0.541–0.897) 0.792 (0.629–0.955) 0.861 (0.726–0.995) 0.799 (0.638–0.961)

No significant difference was found in the Kaplan–Meier analysis of survival according to patient gender (*p* = 0.837).

In the Cox regression analysis performed to investigate the effect of texture analysis parameters on predicting death, the only statistically significant parameter was Sum Entropy T1 Post-treatment (*p* = 0.039; Odds Ratio = 0.000 (95% CI: 0.000-0.651).

**Fig. 3:**
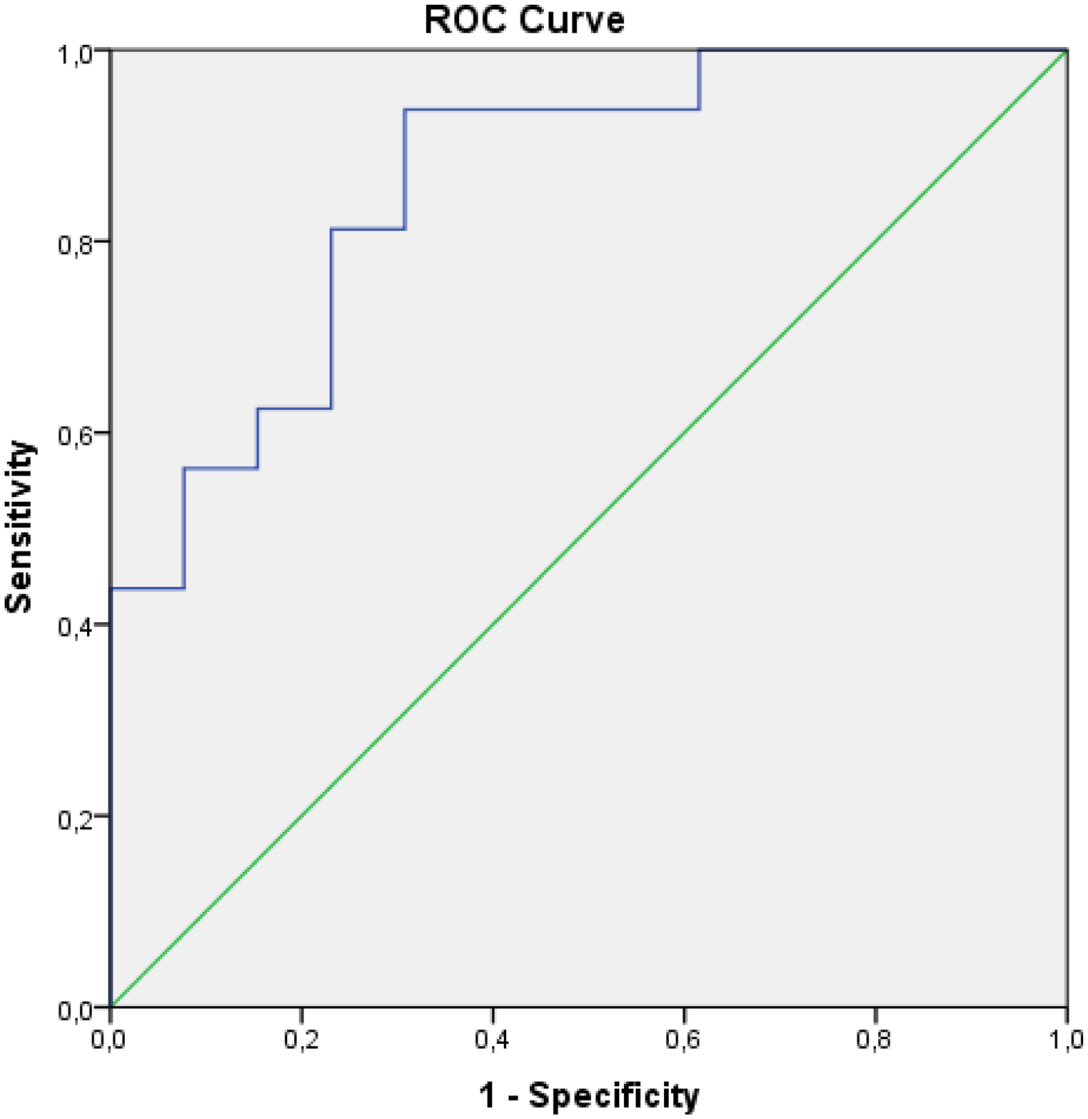
ROC curve for Delta SumEntropy T1 in predicting ≥ 12-month survival (AUC: 0.861; 95% CI: 0.726–0.995)

**Fig. 4:**
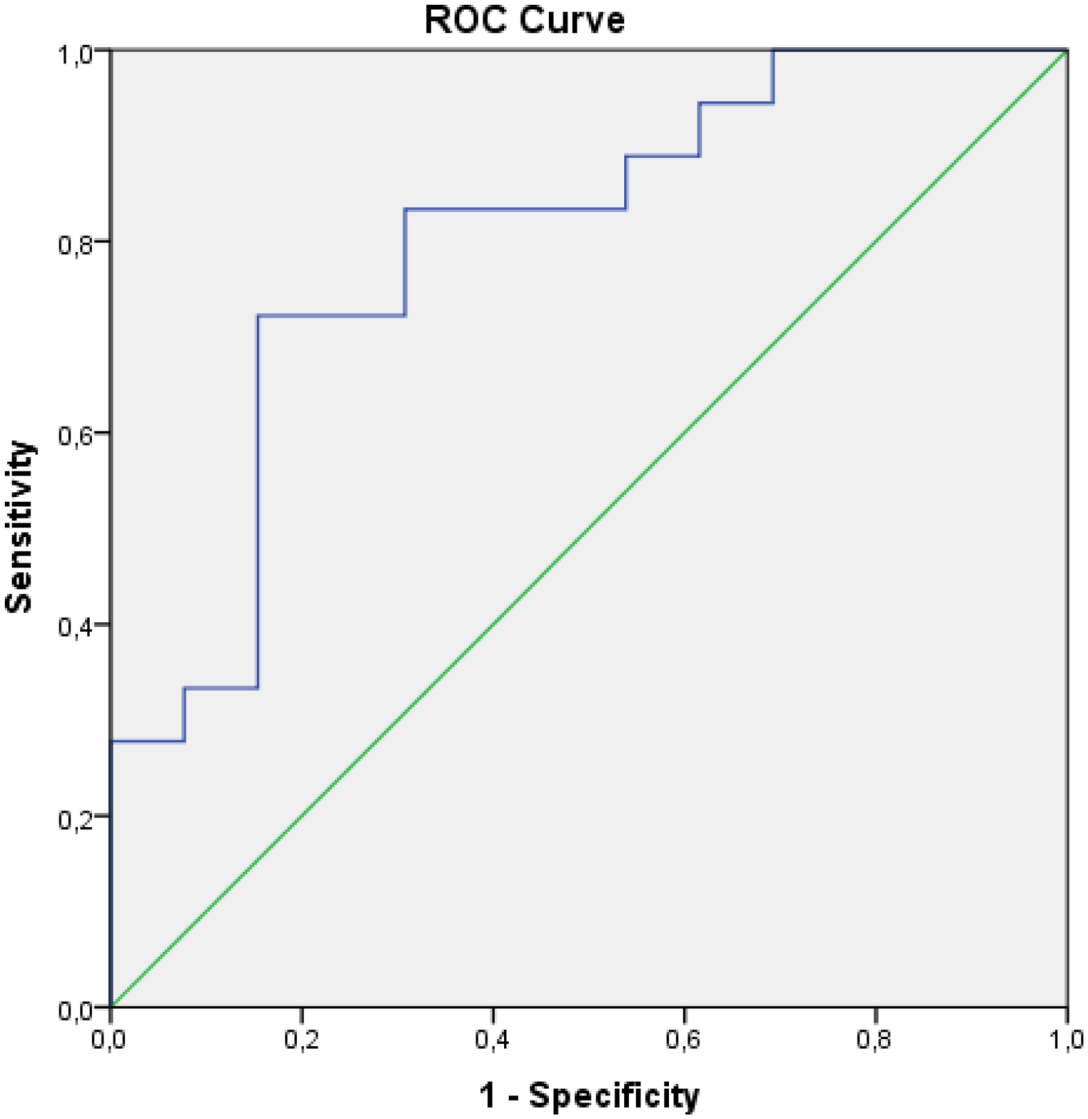
ROC curve for Delta SumEntropy T2 in predicting ≥ 12-month survival (AUC: 0.799; 95% CI: 0.638–0.961)

**Fig. 5:**
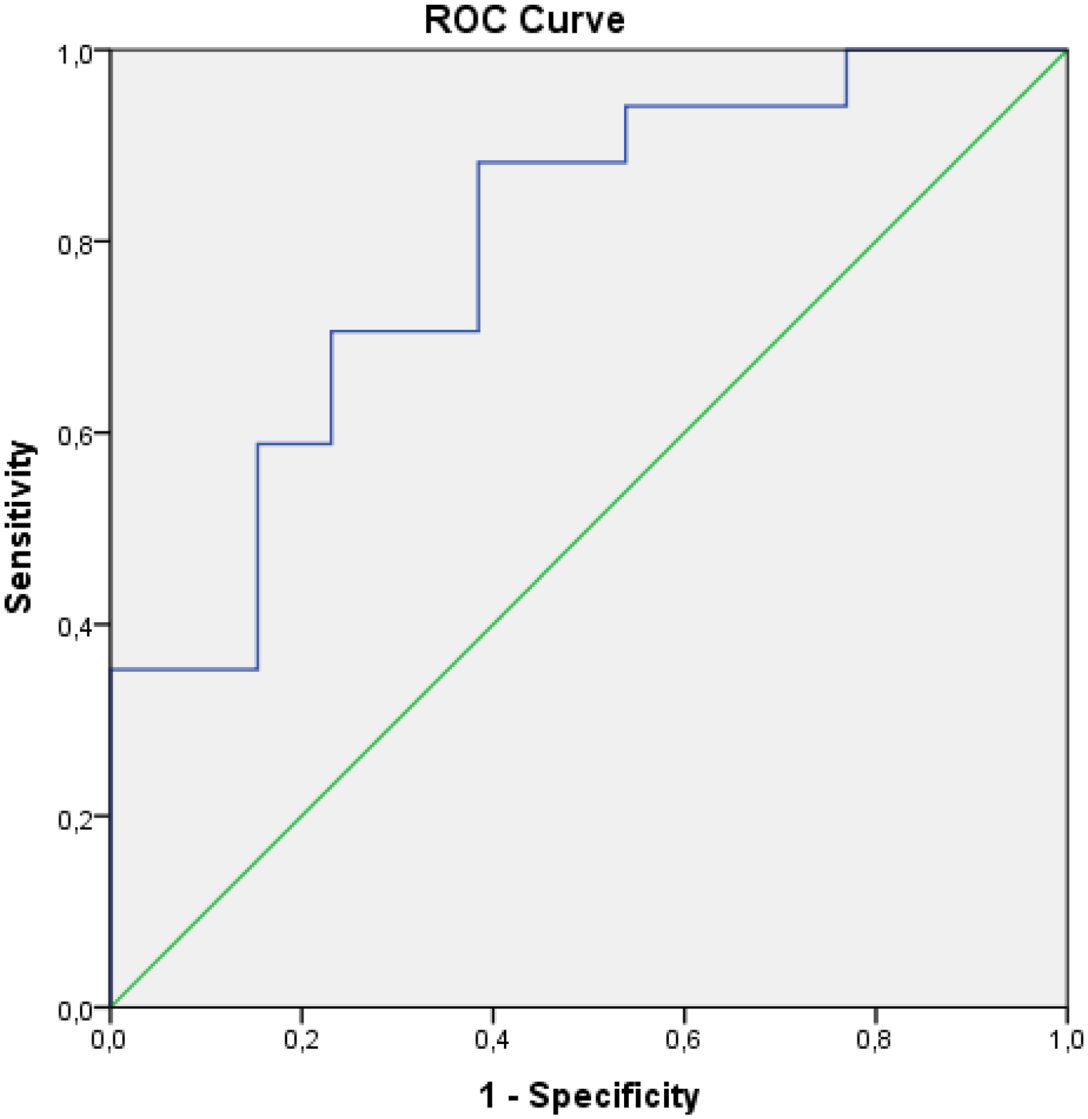
ROC curve for post-treatment SumEntropy T1 in predicting ≥ 12-month survival (AUC: 0.792; 95% CI: 0.629–0.955)

According to the results of ROC analysis evaluating the diagnostic performance in predicting cases with 12 months and more survival, Sum Entropy T1 post-treatment (AUC: 0.792; CI: 0.629–0.955; *p* = 0.007; cut-off = 1.812; sensitivity 88.2%; specificity 61.5%), Delta Sum Entropy T1 (AUC: 0.861; CI: 0.726–0.995; *p* = 0.001; cut-off = 0.981; sensitivity 93.8%; specificity 69.2%), Delta Sum Entropy T2 (AUC: 0.799; CI: 0.638–0.961; *p* = 0.005; cut-off = 0.992; sensitivity 83.3%; specificity 69.2%) parameters were found to be statistically significant (Figs. 3 and 4-5). According to the ROC analysis results evaluating the diagnostic performance in predicting cases with survival under 12 months, the Sum Average T1 pre-treatment parameter (AUC: 0.719; CI: 0.541–0.897; *p* = 0.030; cut-off = 63.7; sensitivity 75%; specificity 66.7) was found to be statistically significant.

## Discussion

PDMGs have bleak prognosis, with a median overall survival of approximately 1 year. Previous studies have explored various imaging methods, such as MR perfusion, MR spectroscopy, and texture analysis, to estimate overall survival [7, 11–14, 20]. Estimation of prognosis may influence treatment planning and provide appropriate information to patients and their families. In our study, we investigated delta radiomics, which enables the evaluation of temporal changes in tumor imaging characteristics, and assessed its potential to predict overall survival in children with PDMG.

In our cohort, the median overall survival was 13 months, which is quite similar to the results reported by Tam et al. (11 months) [13] and Liu et al. (12 months) [11]. In contrast, Szychot et al [14]. described a shorter survival of 9.6 months, whereas Hipp et al. and Chilaca-Rosas et al. each reported longer survival times of approximately 15 months [20, 21]. Taken together, these results indicate that our findings are in line with multicenter and radiomics-based studies, and underscore that PDMGs are associated with a poor prognosis despite such differences.

In the study by Leach et al., several conventional MRI features were linked to reduced overall survival, including extrapontie extension, larger tumor size, occurrence of necrosis, enhancement, or diffusion restriction, and distant disease at diagnosis [5]. Nevertheless, Sedlacik et al. found no significant association between tumor volume and OS [22]. Otherwise, increased Cho: NAA ratio and metabolic heterogeneity on MR spectroscopy [23], low ADC values on diffusion weighted imaging [24], and reduced tumor perfusion parameters [22] have each been considered indicators of poor prognosis. However, Sedlacik et al. highlighted that ASL and DSC perfusion methods have technical limitations that may affect measurement reliability [22]. Similarly, the use of MRS in PDMG is constrained by its intrinsically low spatial resolution and the technical challenges of acquiring images in the posterior fossa [25]. For these reasons, there is a need for relatively more feasible and non-invasive stratification methods.

Recent radiomics studies have produced contradictory results regarding the prognostic impact of tumor heterogeneity in PDMGs. In a small biopsy-proven H3K27M-mutant cohort (*n* = 12), baseline MRI features indicating higher intra-tumoral heterogeneity (e.g., rim variability, peak intensity parameters) were related to worse OS [21]. In contrast, Szychot et al. stated that the mean of positive pixels derived from T2-WI was the strongest prognostic marker of overall survival, with more homogeneous tumor texture predicting poorer survival [14]. Similarly, Liu et al. found that baseline tumor homogeneity was linked to poor prognosis, and Wagner et al. highlighted that voxel-level texture features capturing intra-tumoral heterogeneity, particularly gray level size zone and run length, were the strongest predictors of PFS at 4 months, with greater heterogeneity associated with better prognosis [11, 12]. Tam et al. also analyzed texture features derived from post-contrast T1 and T2-WI and, in line with these findings, demonstrated that greater heterogeneity correlated with better prognosis [13]. In our study, the only significant baseline texture feature was the sum average derived from non-contrast T1-weighted MRI. Lower values may reflect low-intensity regions such as necrotic or hypocellular areas and were associated with better prognosis.

In contrast to baseline imaging, available data on post-RT texture analysis in PDMGs remain scarce. Liu et al. reported that post-contrast T1-derived features, particularly a lower 10th percentile gray level value—which may reflect necrotic areas within the tumor core—were associated with longer OS [11]. On the contrary, Szychot et al. did not find any association between prognosis and post-RT texture features [14]. Our results, consistent with Liu et al.’s findings, suggest that higher post-RT SumEntropy values predict longer OS, which may reflect intratumoral heterogeneity or necrosis.

According to available data, this is the first study to report on the role of delta radiomics features for prognostic prediction in PDMGs. In other malignancies, such as locally advanced rectal cancer, delta radiomics has been shown to outperform clinical parameters for predicting distant metastasis risk, and in colorectal liver metastases, CT-based delta radiomics combined with clinical factors provided the highest performance in progression-free survival [15, 26]. Additionally, delta radiomics revealed superior prognostic value in estimating OS in recurrent glioblastoma patients [27]. In our study, delta radiomics SumEntropy also yielded significant AUC values, which were higher than those of post-RT SumEntropy alone. This may indicate that increased heterogeneity after RT is associated with longer OS and could reflect a more distinct intratumoral biological response to radiotherapy, suggesting that higher delta SumEntropy values may similarly correlate with improved overall survival. Consistently, in a glioblastoma mouse model, delta radiomics was reported to detect radiation-induced intratumoral microstructural changes earlier than single-time-point radiomics, suggesting its potential as a non-invasive method for treatment response assessment [18].

Recent work in pediatric DMG imaging has emphasized that AI-supported analysis can help organize complex multimodal data and reduce variability in quantitative imaging [25]. Building on this concept, the integration of delta radiomics into automated AI pipelines could further simplify longitudinal assessment by comparing pre- and post-treatment features without manual intervention. Such an approach has the potential to make temporal radiomic analysis more practical in clinical and research settings, supporting more consistent follow-up evaluations and potentially improving the feasibility of non-invasive prognostic modeling.

Our institution does not currently have a PACS-integrated radiomics platform, literature suggests that such systems—such as SQLite4Radiomics—could enable the practical implementation of radiomics in daily clinical workflow. The development of PACS-integrated radiomics platforms, such as SQLite4Radiomics, indicates that fully automated radiomic feature extraction within standard clinical workflows is becoming feasible, potentially facilitating the future clinical adoption of delta-radiomics and enhancing the contribution of radiologists to patient management and prognostication [28]. 

Our study has several limitations that need to be considered when interpreting the results. Firstly, the study design was retrospective and included a limited, single-center cohort, which may affect reproducibility and increase the risk of overfitting; thus, external validation in larger multi-center studies is warranted. Secondly, patients were diagnosed on a clinico-radiological basis, and molecular or mutational analyses were not performed. It is well established that mutation status, such as H3K27M, influences prognosis in PDMG patients. While biopsy has been reported to be safe for diagnostic purposes, repeat biopsy to evaluate treatment response is not feasible. Thirdly, pre- and post-treatment MRI scans were obtained using different scanners within routine clinical workflow, which may have introduced variability in image acquisition and radiomic feature stability. In addition, inter-observer or intra-observer variability was not assessed, and manual segmentation may be associated with inherent subjectivity. Lastly, manual segmentation and the use of software not integrated with the PACS system are time-consuming and may limit the clinical applicability of the method.

## Conclusion

PDMGs remain among the pediatric tumors with the poorest prognosis. Our preliminary results suggest that delta radiomics has superiority in estimating OS and may be useful for prognostic stratification in patients with PDMGs. Early prognostic assessment could also facilitate the planning of personalized treatment strategies. Multi-center and prospective studies with external validation are warranted to confirm the potential of delta radiomics for OS prediction.

## Supplementary Information

Below is the link to the electronic supplementary material.

Supplementary Material 1

## Data Availability

No datasets were generated or analysed during the current study.
