# Supplementary material for: Prognostic role of delta radiomics in pediatric pontine diffuse midline gliomas
Source: J Neurooncol. 2026 Feb 11;176(3):206. doi: 10.1007/s11060-026-05457-y (PMC12894174; doi:10.1007/s11060-026-05457-y)
Supplement: Supplementary file 1 — Supplementary Material 1 [file 11060_2026_5457_MOESM1_ESM.docx]

|  | **p values for predicting survival** | | |
| --- | --- | --- | --- |
| **Texture features** | **ADC** | **Precontrast T1W** | **T2W** |
| Mean pre-treatment | 0,195 | 0,679 | 0,596 |
| Mean post-treatment | 0,472 | 0,950 | 0,779 |
| Delta Mean | 0,889 | 0,203 | 0,660 |
| Variance pre-treatment | 0,666 | 0,836 | 0,766 |
| Variance post-treatment | 0,767 | 0,884 | 0,496 |
| Delta Variance | 0,610 | 0,357 | 0,078 |
| Skewness pre-treatment | 0,540 | 0,890 | 0,305 |
| Skewness post-treatment | 0,582 | 0,267 | 0,050 |
| Delta Skewness | 0,353 | 0,483 | 0,968 |
| Kurtosis pre-treatment | 0,614 | 0,809 | 0,947 |
| Kurtosis post-treatment | 0,330 | 0,346 | 0,262 |
| Delta Kurtosis | 0,507 | 0,759 | 0,873 |
| Perc. 01% pre-treatment | 0,829 | 0,782 | 0,608 |
| Perc. 01% post-treatment | 0,271 | 0,490 | 0,704 |
| Delta Perc. 01% | 0,472 | 0,483 | 0,873 |
| Perc. 10% pre-treatment | 0,746 | 0,756 | 0,573 |
| Perc. 10% post-treatment | 0,219 | 0,630 | 0,873 |
| Delta Perc. 10% | 0,676 | 0,380 | 1,000 |
| Perc. 50% pre-treatment | 0,428 | 0,730 | 0,655 |
| Perc. 50% post-treatment | 0,409 | 0,851 | 0,749 |
| Delta Perc. 50% | 0,486 | 0,254 | 0,749 |
| Perc. 90% pre-treatment | 0,097 | 0,523 | 0,305 |
| Perc. 90% post-treatment | 0,472 | 0,900 | 0,968 |
| Delta Perc. 90% | 0,430 | 0,254 | 0,936 |
| Perc. 99% pre-treatment | 0,207 | 0,557 | 0,320 |
| Perc. 99% post-treatment | 0,832 | 0,917 | 0,603 |
| Delta Perc. 99% | 0,178 | 0,254 | 0,603 |
| AngScMom pre-treatment | 0,746 | 0,629 | 0,619 |
| AngScMom post-treatment | 0,767 | 0,439 | 0,118 |
| Delta AngScMom | 0,963 | 0,759 | 0,093 |
| Contrast pre-treatment | 0,078 | 0,285 | 0,921 |
| Contrast post-treatment | 0,525 | 0,161 | 0,841 |
| Delta Contrast | 0,265 | 0,136 | 0,936 |
| Correlation pre-treatment | 0,249 | 0,285 | 0,691 |
| Correlation post-treatment | 0,611 | 0,137 | 0,749 |
| Delta Correlation | 0,853 | 0,148 | 0,575 |
| SumOfSqs pre-treatment | 0,280 | 0,836 | 0,197 |
| SumOfSqs post-treatment | 0,310 | 0,660 | 0,317 |
| Delta SumOfSqs | 0,378 | 0,599 | 0,660 |
| InvDfMom pre-treatment | 0,249 | 0,214 | 0,667 |
| InvDfMom post-treatment | 0,966 | 0,630 | 0,317 |
| Delta InvDfMom | 0,378 | 0,219 | 0,496 |
| SumAverg pre-treatment | 0,494 | **0,030** | 0,487 |
| SumAverg post-treatment | 0,498 | 0,217 | 0,689 |
| Delta SumAverg | 0,577 | 0,054 | 0,173 |
| SumVarnc pre-treatment | 0,640 | 0,469 | 0,175 |
| SumVarnc post-treatment | 0,899 | 0,174 | 0,522 |
| Delta SumVarnc | 0,676 | 0,188 | 0,496 |
| SumEntrp pre-treatment | 0,368 | 0,105 | 0,289 |
| SumEntrp post-treatment | 1,00 | **0,007** | 0,101 |
| Delta SumEntrp | 0,963 | **0,001** | **0,005** |
| Entropy pre-treatment | 0,692 | 0,945 | 0,691 |
| Entropy post-treatment | 0,799 | 0,325 | 0,139 |
| Delta Entropy | 0,963 | 0,456 | 0,050 |
| DifVarnc pre-treatment | 0,072 | 0,285 | 0,921 |
| DifVarnc post-treatment | 0,899 | 0,063 | 0,873 |
| Delta DifVarnc | 0,086 | 0,136 | 0,904 |
| DifEntrp pre-treatment | 0,150 | 0,241 | 0,843 |
| DifEntrp post-treatment | 0,799 | 0,217 | 0,447 |
| Delta DifEntrp | 0,246 | 0,161 | 0,575 |

**Supplementary Table 1.** Complete list of all extracted texture features used in the analysis.
